# COL19A1 is a predictive biomarker for the responsiveness of esophageal squamous cell carcinoma patients to immune checkpoint therapy

**DOI:** 10.1111/1759-7714.14873

**Published:** 2023-04-02

**Authors:** Jian‐Hua Liu, Ju‐Ze Lin, Qianhui Qiu, Changbin Zhu, Weiwei Li, Qian Li, Zhan Huang, Xueer Xia, Guibin Qiao, Jiming Tang

**Affiliations:** ^1^ Department of Oncology, Guangdong Provincial People's Hospital (Guangdong Academy of Medical Sciences) Southern Medical University 510080 Guangzhou China; ^2^ Ganzhou Hospital of Guangdong Provincial People's Hospital Ganzhou Municipal Hospital Ganzhou Jiangxi China; ^3^ Department of Traditional Chinese Medicine, Guangdong Provincial People's Hospital (Guangdong Academy of Medical Sciences) Southern Medical University 510080 Guangzhou China; ^4^ Department of Otolaryngology‐Head and Neck Surgery, Guangdong Provincial People's Hospital (Guangdong Academy of Medical Sciences) Southern Medical University 510080 Guangzhou China; ^5^ Department of Translational Medicine Amoy Diagnostics Co., Ltd Xiamen 361027 China; ^6^ Department of Gastrointestinal Surgery, General Surgery Center, Zhujiang Hospital Southern Medical University Guangzhou 510000 China; ^7^ Department of Thoracic Surgery, Guangdong Provincial People's Hospital (Guangdong Academy of Medical Sciences) Southern Medical University 510080 Guangzhou China

**Keywords:** B cell infiltration, *COL19A1*, esophageal squamous cell carcinoma, neoadjuvant immunotherapy

## Abstract

**Background:**

The use of neoadjuvant immunotherapy plus chemotherapy has revolutionized the management of esophageal squamous cell carcinoma (ESCC) patients. Nevertheless, patients who would maximally benefit from these therapies have not been identified.

**Methods:**

We collected postoperative specimens from 103 ESCC patients, of which 66 patients comprised a retrospective cohort and 37 comprised a prospective cohort. Patient specimens were subjected to applied multi‐omics analysis to uncover the mechanistic basis for patient responsiveness to cancer immunotherapy. The tumor microenvironment characteristics of these patient specimens was explored and identified by multiplex immunofluorescence and immunohistochemistry.

**Results:**

Results demonstrated high *COL19A1* expression to be a novel biomarker for successful immunotherapy (*COL19A1* ^high^, odds ratio [95% confidence interval]: 0.31 [0.10–0.97], *p* = 0.044). Compared with *COL19A1* ^low^ patients, *COL19A1* ^high^ patients benefited more from neoadjuvant immunotherapy (*p* < 0.01), obtained better major pathological remissions (63.3%, *p* < 0.01), with a trend toward better recurrence‐free survival (*p* = 0.013), and overall survival (*p* = 0.056). Moreover, analysis of an immune‐activation subtype of patients demonstrated increased B cell infiltration to be associated with favorable patient survival and a better response to neoadjuvant immunotherapy plus chemotherapy.

**Conclusions:**

The findings of this study provide insight into the optimal design of individual treatments for ESCC patients.

## Graphical abstract

A flowchart of the study showed that high *COL19A1* expression conferred a good prognosis for esophageal squamous cell carcinoma (ESCC) patients, and it was also a novel biomarker for successful immunotherapy. Moreover, analysis of the immune‐activation subtype demonstrated different distribution of 29 types of immune related signatures in the retrospective cohort.

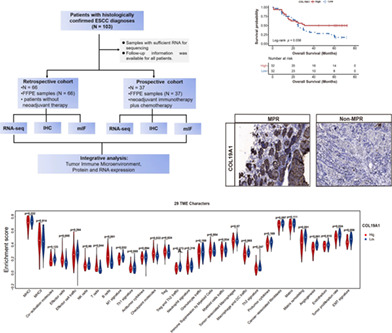

## INTRODUCTION

Esophageal cancer (EA) is the sixth most frequently diagnosed cancer type and the seventh leading cause of tumor‐associated mortality worldwide.^1^ As the predominant histological subtype, esophageal squamous cell carcinoma (ESCC) is characterized by an aggressive nature, accounting for approximately 90% of EA cases in China.^2^ Due to an absence of specific symptoms for early ESCC, most patients have advanced disease at initial diagnosis. Although significant treatment progress has been accomplished by combination therapy (chemotherapy, targeted therapy, radiotherapy, and surgery), survival of patients with ESCC remains dismal, with a 5‐year survival ≤50%.^3^ Further, the efficacy of conventional chemotherapy has plateaued due to the development of tumor resistance to the therapeutic agent and the inherent toxicity of the agent. Therefore, there is an urgent need to develop revolutionary treatments for ESCC.

Currently, the use of antiprogrammed death 1 (PD‐1)/programmed death‐ligand 1 (PD‐L1) antibodies has been a striking success. The antibodies are immune checkpoint inhibitors (ICIs) that boost host immunity. The use of these antibodies represents a novel approach to drug development and precision medicine.^4^, ^5^ It is well known that ESCC patients can benefit remarkably from first‐, second‐line, and perioperative immunotherapy.^6^, ^7^, ^8^, ^9^, ^10^ For locally advanced ESCC, accumulating evidence demonstrates neoadjuvant immunotherapy combined with chemotherapy and surgery, results in better tumor regression and R0 resection rates than chemotherapy alone.^11^, ^12^, ^13^ Our previous study confirmed the promising efficacy and good safety of ICIs in patients with resectable ESCC.^14^ Further, there are ongoing clinical trials of these forms of neoadjuvant immunotherapy, such as JCOG1804E and Palace‐1.^15^, ^16^

However, some ESCC patients fail to benefit from ICIs, with the reason for these failures unclear. Accumulating evidence indicates that the tumor immune microenvironment (TIME) is key to tumor‐immune interactions and the response to therapy.^17^, ^18^, ^19^ TIME is influenced by many factors including tumor mutations, protein overexpression, cytokines, and inflammation, among others.^18^, ^19^, ^20^ ESCC has a high degree of heterogeneity, with the tumor microenvironment (TME) of ESCC and its interaction with immunotherapy poorly understood. It is therefore imperative to explore the immune response within the ESCC TME and also to observe resultant clinical outcomes. At present, biomarkers related to the efficacy of immunotherapy are the expression level of PD‐L1,^21^ mismatch repair‐deficient/high microsatellite instability,^22^ and tumor mutation burden (TMB).^23^, ^24^ To date, there has been no comprehensive transcriptomic analysis of the intertumor complexity of ESCC and the response of ESCC to neoadjuvant immunotherapy.

Herein, we classified ESCC based on transcriptomics and subtype‐specific TME characteristics. *COL19A1*, a novel biomarker overexpressed in an immune‐enriched subtype, was found to be associated with the best overall survival and with a major pathological response (MPR) following neoadjuvant immunotherapy. Based on these results, an immune‐related prediction model for prognosis and response to immunotherapy was successfully established. The model allows for exploration of the immune‐activated ESCC landscape. The use of the model will aid development of immune therapies for ESCC patients.

## METHODS

### Patient cohorts

Two independent patient cohorts (retrospective cohort [*N* = 66, treatment‐naive samples] and prospective cohort [*N* = 37, received neoadjuvant immunotherapy plus chemotherapy]) from Guangdong Provincial People's Hospital (Guangzhou, China) were enrolled in this study. In all, 103 paraffin‐embedded ESCC specimens, including the deepest lesions, from January 2015 to May 2021 were prepared for RNA sequencing. The clinical information of each participant was obtained from medical records. Clinical stage was classified or reclassified according to the American Joint Committee of Cancer eighth edition ESCC staging system.^3^ Progression‐free survival (PFS), recurrence‐free survival (RFS), overall survival (OS), and MPR were all defined according to the Response Evaluation Criteria in Solid tumors version 1.1 (RECIST 1.1). The cutoff date for the last follow‐up was February 2022. The study was approved by the Medical Ethics Committee of Guangdong Provincial People's Hospital.

### RNA sequencing

Total RNA was extracted according to the manufacturer's instructions. RNA quantity, quality, and integrity was assessed, with an RNA integrity score >6.0 considered satisfactory. RNA was fragmented in accordance with the DV200 value. After evaluation of fragment length, reverse transcription and complementary DNA synthesis of RNA fragments were performed, followed by preparation of a strand‐specific library.

Prepared libraries were sequenced with an Illumina Nova Seq 6000 instrument (Illumina) using 2 × 150 bp paired‐end reads based on established experimental quality control parameters. Raw sequencing data were then converted to fastq format (Illumina), as described in our recent study.^14^

### Consensus clustering

Unsupervised consensus clustering, a class discovery approach, is used to identify unknown possible subtypes that present similar intrinsic features according to differences in gene expression profiles.^25^ In this study, after 80% of the specimens were sequentially extracted 100 times, 5000 highly variable expressed genes were applied for hierarchical clustering analysis. Results were based on construction of a cumulative distribution function (CDF) in accordance with consensus matrices.

### Differentially expressed gene (DEG) selection and pathway analysis

RNA sequencing (RNA‐seq) expression data of the two cohorts were transformed into transcripts per million (TPM) for analysis. DEGs were defined based on specific criteria as follows; specific subtype versus other subtypes, genes with Log_2_FC ≥1, and *p* < 0.05. Heatmaps and volcano diagrams were constructed to visualize DEGs.

Differential gene expression analysis was performed using the EdgeR package, with major criteria as previously described.^14^ Enrichment analyses were performed to determine gene ontology (GO) and Kyoto Encyclopedia of Genes and Genomes (KEGG) pathways.

### Evaluation of infiltrating immune cells in the TME

Based on the transcriptome data of the two independent cohorts, characteristics of the ESCC TME were identified. The relative enrichment of infiltrating immune cells was evaluated using a single‐sample gene set enrichment analysis (ssGSEA) algorithm. Twenty‐eight immune cell types and 29 types of immune‐related signatures^26^ were assessed to calculate the relative proportion of immune cell types.

### Immunohistochemistry (IHC) and multiplex immunofluorescence (mIF)

To validate results, IHC and mIF were carried out following recommended protocols.^27^ For IHC analysis, formalin‐fixed paraffin‐embedded (FFPE) tumor specimens were stained with primary antibodies reactive with; Ki‐67, PD‐L1, and COL19A1. The Akoya Opal 5‐color fluorescent detection platform was used to identify distinctive ESCC TME characteristics. Tissues were simultaneously examined with an Opal Polaris 5‐Color Automation IHC Kit. Image acquisition and signal quantification (CD8, CD20, CD27, and DAPI) were performed using the Panoramic MIDI Pathology Imaging System. For quality control purposes, each analyzed image was reviewed independently, with histological evaluations performed independently by two blinded pathologists. Detailed antibody information is provided in Appendix S1, Supplementary Materials.

### Statistical analysis

The Kaplan–Meier method was used to determine survival outcomes (RFS and OS). Differences in variables were compared using the Kruskal–Wallis test. R package analysis included “Consensus Cluster Profiler (Unsupervised clustering),” “DESeq29 (Differential gene expression),” “ggplot2 (Boxplot),” and “randomForest (Subtype prediction model).” R software (V4.0.5) was used for all bioinformatic analysis. A *p* ≤ 0.05 for the two‐sided Mann–Whitney U test was considered statistically significant.

## RESULTS

### Study population and baseline characteristics

**FIGURE 1:**
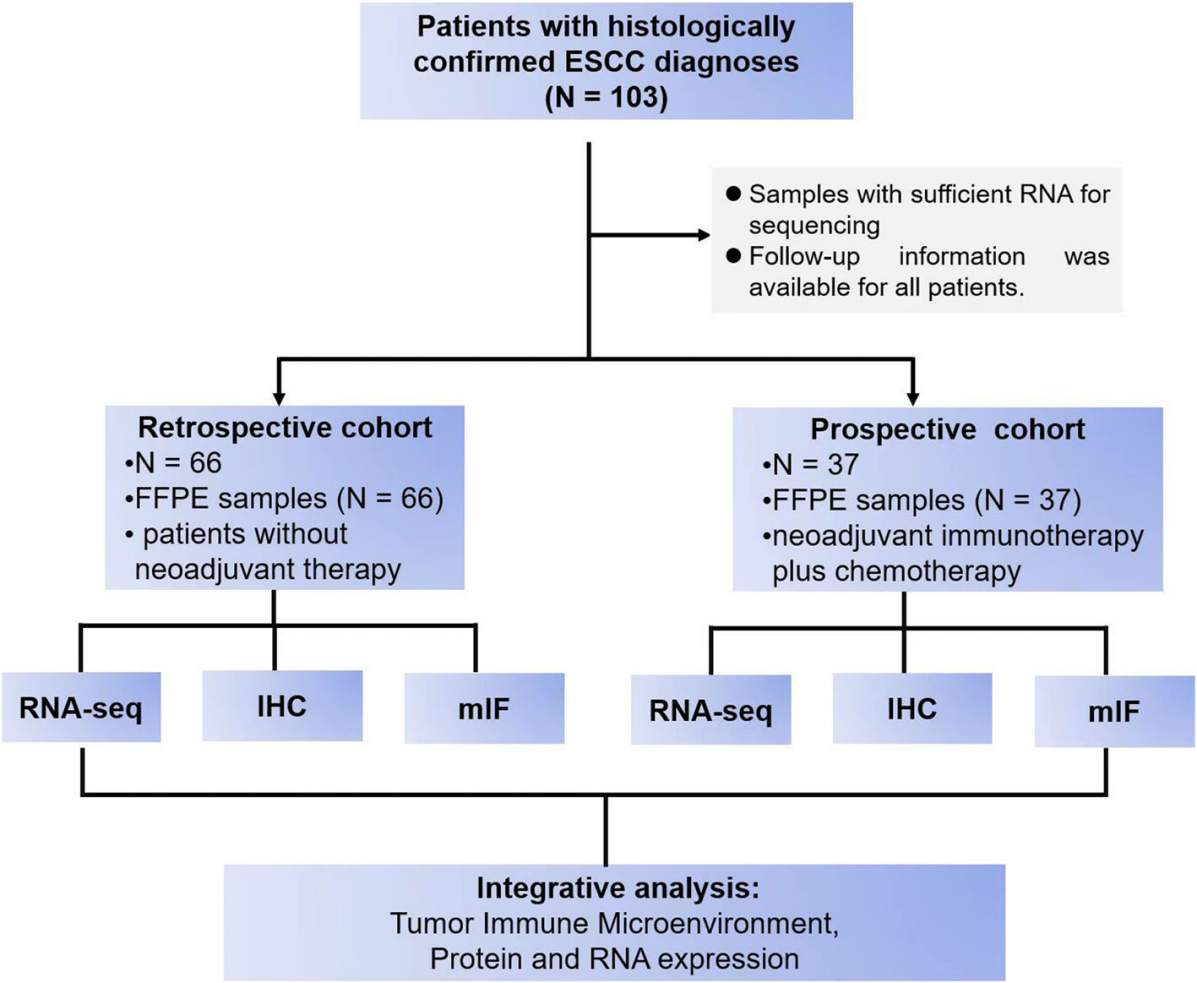
Study profile.

A total of 103 ESCC patients were included in this study, with (*N* = 66) for the retrospective cohort and (*N* = 37) for the prospective cohort. A flowchart of the study is shown in Figure 1. Details of baseline characteristics are summarized in Tables 1 and 2, which have not been previously reported.^14^

**TABLE 1:** Clinicopathological characteristics of the retrospective cohort of esophageal squamous cell carcinoma (ESCC) patients.

Molecular subtypes (mean ± SD/number [%]/mean [range])					
Characteristics	C1	C2	C3	C4	*p*‐value
Cases	24	22	10	10	
Age (years)					0.315
Mean (SD)	62.1 (9.6)	59.6 (9.6)	63.5 (8.8)	57.6 (6.7)	
Range	47.0–82.0	46.0–83.2	46.0–75.0	45.0–69.0	
Sex					0.951
Male	18 (75.0%)	18 (81.8%)	8 (80.0%)	8 (80.0%)	
Female	6 (25.0%)	4 (18.2%)	2 (20.0%)	2 (20.0%)	
Tumor location					0.346
Upper	1 (4.2%)	2 (9.1%)	3 (30.0%)	2 (20.0%)	
Middle	15 (62.5%)	12 (54.5%)	3 (30.0%)	6 (60.0%)	
Lower	8 (33.3%)	8 (36.4%)	4 (40.0%)	2 (20.0%)	
Tumor differentiation					0.648
High	4 (16.7%)	4 (18.2%)	0 (0.0%)	0 (0.0%)	
Middle	12 (50.0%)	12 (54.5&)	6 (60.0%)	6 (60.0%)	
Low	8 (33.3%)	6 (27.3%)	4 (40.0%)	4 (40.0%)	
Pathological T stage					0.859
T1	1 (4.2%)	0 (0.0%)	0 (0.0%)	0 (0.0%)	
T2	10 (41.7%)	9 (40.9%)	3 (30.0%)	3 (30.0%)	
T3	12 (50%)	13 (59.1%)	7 (70.0%)	7 (70.0%)	
T4	1 (4.2%)	0 (0.0%)	0 (0.0%)	0 (0.0%)	
Pathological N stage					0.051
N0	11 (45.8%)	13 (59.1%)	9 (90.0%)	10 (100.0%)	
N1	9 (37.5%)	6 (27.3%)	1 (10.0%)	0 (0.0%)	
N2	4 (16.7%)	3 (13.6%)	0 (0.0%)	0 (0.0%)	
Clinical stage					0.083
II	12 (50.0%)	13 (59.1%)	9 (90.0%)	10 (100.0%)	
IIIA	4 (16.7%)	3 (13.6%)	0 (0.0%)	0 (0.0%)	
IIIB	8 (33.3%)	6 (27.3%)	1 (10.0%)	0 (0.0%)	
Vascular invasion					0.314
No	18 (75.0%)	18 (81.8%)	9 (90.0%)	10 (100.0%)	
Yes	6 (25.0%)	4 (18.2%)	1 (10.0%)	0 (0.0%)	
Neuroinvasion					0.678
No	15 (62.5%)	17 (77.3%)	7 (70.0%)	6 (60.0%)	
Yes	9 (37.5%)	5 (22.7%)	3 (30.0%)	4 (40.0%)	
SUV of primary lesion					0.195
Mean (SD)	10.9 (4.9)	14.7 (4.8)	12.4 (5.4)	12.5 (5.6)	
Range	4.6–26.8	6.5–27.9	4.8–19.5	3.6–21.2	
Overall survival status					0.271
Death	9 (37.5%)	4 (18.2%)	5 (50.0%)	4 (40.0%)	
Alive	15 (62.5%)	18 (81.2%)	5 (50.0%)	6 (60.0%)	
Adjuvant therapy					0.700
NA	17 (70.8%)	16 (72.7%)	6 (60.0%)	8 (80.0%)	
Chemotherapy	6 (25.0%)	5 (22.7%)	2 (20.0%)	1 (10.0%)	
Chemoradiotherapy	1 (4.2%)	1(4.5%)	2 (20.0%)	1 (10.0%)	

*Note*: Kruskal–Wallis test.

Abbreviations: NA, not available; SD, standard deviation; SUV, standardized uptake value.

* Indicates *p* < 0.05.

** Indicates *p* < 0.01.

*** Indicates *p* < 0.001.

**TABLE 2:** Clinicopathological characteristics of the prospective cohort of esophageal squamous cell carcinoma (ESCC) patients.

Characteristics	CI	C2	C3/C4	*p*‐value
Cases	13	19	5	
Age (years)				0.512
Mean (SD)	57.3 (8.5)	60.8 (9.7)	58.4 (8.3)	
Range	40.0–69.0	51.0–70.0	51.0–70.0	
Sex				0.276
Male	12 (92.3%)	15 (78.9%)	3 (60.0%)	
Female	1 (7.7%)	4 (21.1%)	2 (40.0%)	
Tumor location				0.027
Upper	6 (46.2%)	3 (15.8%)	0 (0.0%)	
Middle	7 (53.8%)	8 (42.1%)	2 (40.0%)	
Lower	0 (0.0%)	8 (42.1%)	3 (60.0%)	
Tumor differentiation				0.851
High	2 (15.4%)	2 (10.5%)	0 (0.0%)	
Middle	7 (53.8%)	12 (63.2%)	4 (80.0%)	
Low	4 (30.8%)	5 (26.3%)	1 (20.0%)	
ypT stage				0.358
T0	1 (7.7%)	1 (5.3%)	2 (40.0%)	
T1	2 (15.4%)	3 (15.8%)	0 (0.0%)	
T2	3 (23.1%)	6 (31.6%)	2 (40.0%)	
T3	7 (53.8%)	7 (36.8%)	1 (20.0%)	
T4	0 (0.0%)	2 (10.5%)	0 (0.0%)	
ypN stage				0.641
N0	6 (46.2%)	8 (42.1%)	2 (40.0%)	
N1	4 (30.8%)	5 (26.3%)	2 (40.0%)	
N2	3 (23.1%)	5 (26.3%)	0 (0.0%)	
N3	0 (0.0%)	1 (5.3%)	1 (20.0%)	
Clinical stage				0.110
pCR	1 (7.7%)	1 (5.3%)	2 (40.0%)	
I	2 (15.4%)	5 (26.3%)	1 (20.0%)	
II	5 (38.5%)	2 (10.5%)	0 (0.0%)	
III	5 (38.5%)	10 (52.6%)	1 (20.0%)	
IV	0 (0.0%)	1 (5.3%)	1 (20.0%)	
Vascular invasion				0.092
No	12 (92.3%)	11 (57.9%)	4 (80.0%)	
Yes	1 (7.7%)	8 (42.1%)	1 (20.0%)	
Neuroinvasion				0.081
No	13 (100.0%)	13 (68.4%)	4 (80.0%)	
Yes	0 (0.0%)	6 (31.6%)	1 (20.0%)	
CPS				0.137
≤10	4 (30.8%)	7 (36.8%)	3 (60.0%)	
>10 and ≤50	6 (46.2%)	12 (63.2%)	2 (40.0%)	
>50	3 (23.0%)	0 (0.0%)	0 (0.0%)	

Abbreviations: CPS, combined positive score; pCR, pathological complete response; SD, standard deviation.

* Indicates *p* < 0.05.

** Indicates *p* < 0.01.

*** Indicates *p* < 0.001.

A total of 19 (28.8%) patients in the retrospective cohort received postoperative adjuvant therapy. A total of 22 patients died during the follow‐up period, and all deaths were due to recurrent or progressive disease. All patients in the prospective cohort were treated with the TP regimen (nab‐paclitaxel and cisplatin) combined with a PD‐1 inhibitor (tislelizumab in 29 cases, pembrolizumab in 8 cases). A total of 51.4% of patients achieved MPR, with no patients lost to follow‐up.

### Gene expression profiles demonstrated ESCC patient heterogeneity

Based on the consensus matrix heatmap, patients from the retrospective cohort were clearly divided into four clusters (C1–C4). The percentage of patients with the C1, C2, C3, and C4 subtypes were 36.3% (24/66), 33.3% (22/66), 15.2% (10/66), and 15.2% (10/66), respectively. Each cluster exhibited distinct gene expression and biological properties. Briefly, the C1 subtype exhibited greater enrichment for metabolism‐related genes (metabolism subtype). The C2 subtype mainly exhibited extracellular matrix and mesenchymal characteristics (mesenchymal subtype). The C3 subtype was characterized by immune activation (immune subtype), and the signature genes of the C4 subtype were involved in epithelial cell function (epithelial subtype) (Figure S1A).

The distributions of clinical stage, lymph node (N) stage, tumor differentiation, and other characteristics among the four clusters were compared (Table 1). Results demonstrated molecular subtypes to be significantly associated with clinical stage and pN stage. Patients with the C3 and C4 subtypes tended to have more localized tumor lesions (Figure S1B‐E). Moreover, patients with these two subtypes had a lower incidence of vascular invasion and a slightly higher incidence of neuro‐invasion than those with the other subtypes (Figure S1F‐G). In contrast, in the prospective cohort, 35.1% (13/37) of patients were classified as the C1 subtype, 51.4% (19/37) as the C2 subtype, and 13.5% (5/37) as the C3/4 subtype (including four cases of the C3 subtype and one case of the C4 subtype). These patients were combined for further analysis due to the similarity of immune profiles.

### ***COL19A1*** ^**high**^ **expression was associated with improved responsiveness to immunotherapy and better ESCC patient prognosis**

**FIGURE 2:**
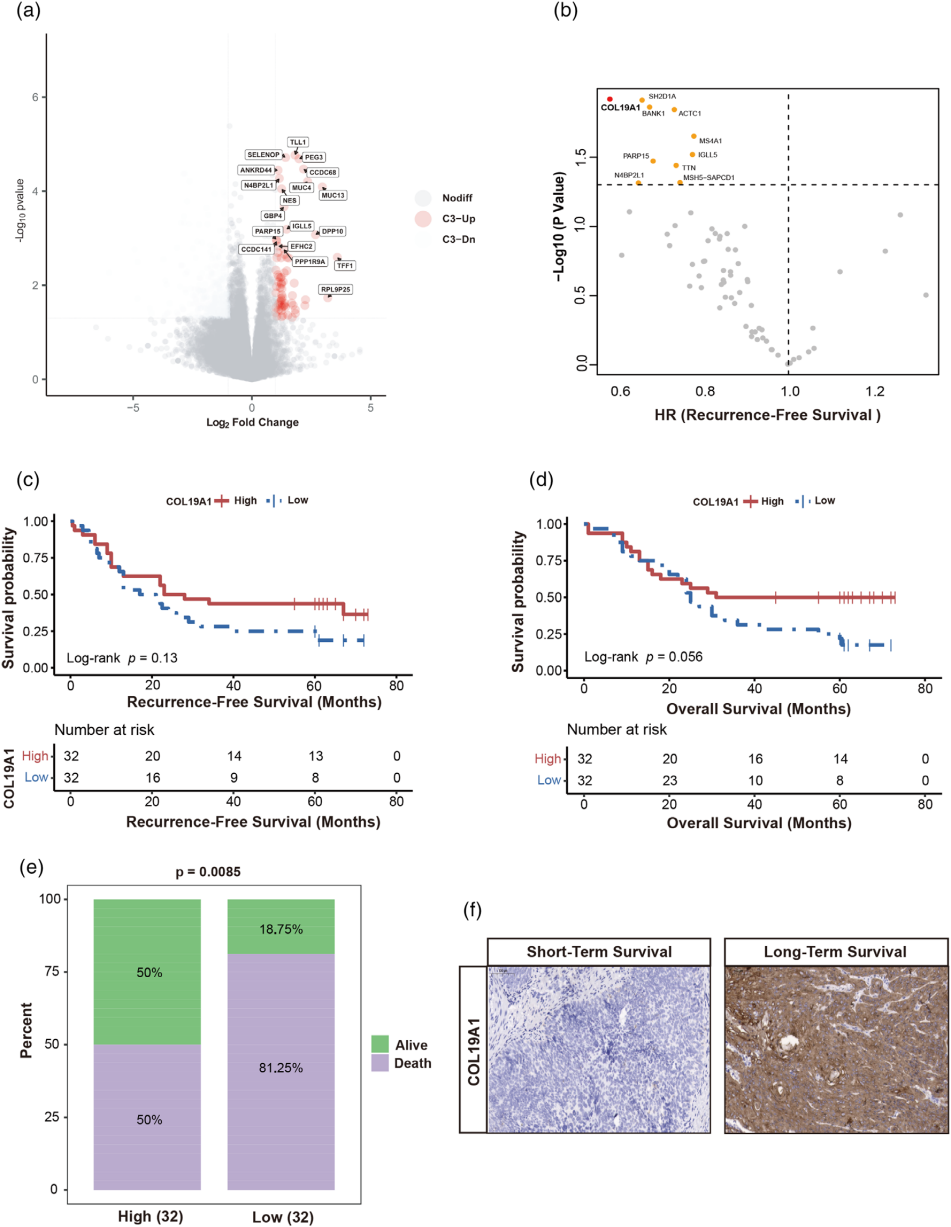
High *COL19A1* expression conferred a good prognosis for ESCC patients. (a) Differential gene expression analysis based on RFS (log_2_FC > 1，padj < 0.1). Red points represent genes with an FDR‐adjusted. (b) Univariate Cox regression analysis was performed to identify six prognostic genes. HR and *p* denoted hazard ratios and *p*‐values, respectively. (c) and (d) Comparison of RFS and OS between *COL19A1* ^low^ and *COL19A1* ^high^ expression subtypes in the retrospective cohort. (e) The immunohistochemistry results of low/high‐*COL19A1* expression subgroups in the retrospective cohort. IO, immunotherapy; non‐MPR, nonmajor pathological remissions; OS, overall survival; RFS, recurrence‐free survival.

In the retrospective cohort, survival analysis demonstrated patients with the C3 subtype to have a more favorable OS than those with the other subtypes. Survival curve characterization and cluster analysis have been previously discussed in a separate publication.^14^ We observed that six upregulated genes were related to longer RFS for C3 subtype patients of the retrospective cohort (Figure 2a, b). *COL19A1* has seldom been investigated in ESCC patients and was selected for further analysis. Patients with high *COL19A1* expression had better RFS (*p* = 0.013) (Figure 2c) and OS (*p* = 0.056) (Figure 2d). The mortality of *COL19A1* ^low^ patients was significantly greater (*p* < 0.01) than that of *COL19A1* ^high^ patients (Figure 2e). IHC staining demonstrated *COL19A1*, which is expressed by cancerous epithelial cells, to be upregulated in ESCC samples from patients with good outcomes (Figure 2f).

**FIGURE 3:**
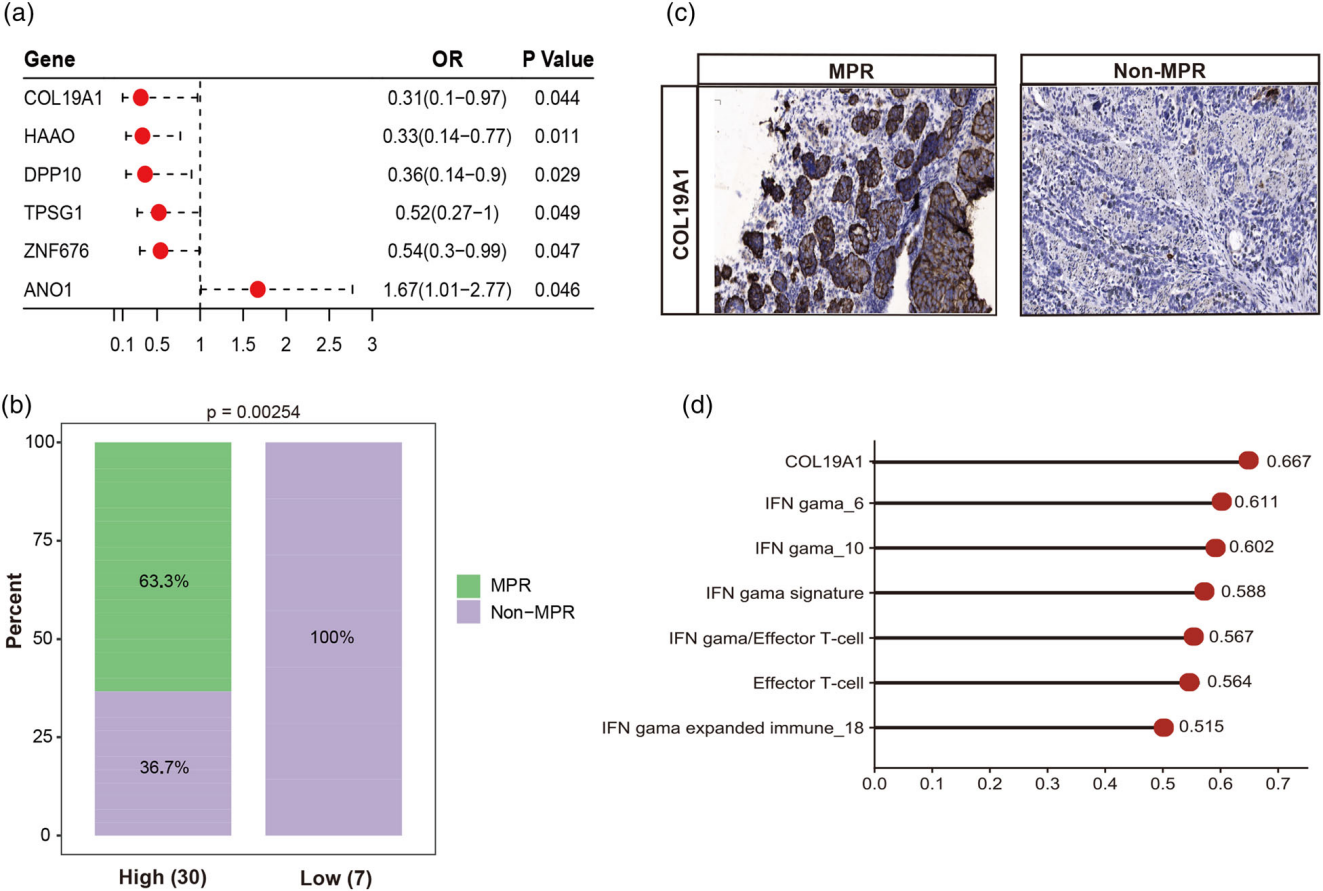
High *COL19A1* expression conferred benefit from anti‐PD‐1 antibody treatment of esophageal squamous cell carcinoma (ESCC) patients. (b) Logistic regression model for differential gene expression for six genes independently associated with a good prognosis. (b) The distribution of non‐MPR and MPR patients among the two different groups. (c) The immunohistochemistry results of low/high‐*COL19A1* expression subgroups in the prospective cohort. (d) AUC values of six genes. AUC, area under curve; IO, immunotherapy; MPR, major pathological remissions.

In the prospective cohort, within the range of subtype‐specific upregulated genes, high *COL19A1* expression was observed to be a novel biomarker that increased the efficacy of anti‐PD‐1 therapy (*COL19A1* ^high^, OR [95% CI]: 0.31 [0.10–0.97], *p* = 0.044) (Figure 3a). Compared with *COL19A1* ^low^ patients (10/18, 27.8%), *COL19A1* ^high^ patients (30/37, 63.3%) benefited more from neoadjuvant chemoimmunotherapy (*p* < 0.01) and achieved a significantly higher MPR (*p* < 0.01) (Figure 3b). Moreover, IHC findings corroborated these conclusions at the protein level (Figure 3c). Further, *COL19A1* ^high^ expression was found to be an independent prognostic factor for a superior clinical outcome compared with published immunotherapy predictive biomarkers (the area under the curve was 0.667) (Figure 3d).

### ***COL19A1*** ^**high**^ **expression was associated with ESCC immune activation**

**FIGURE 4:**
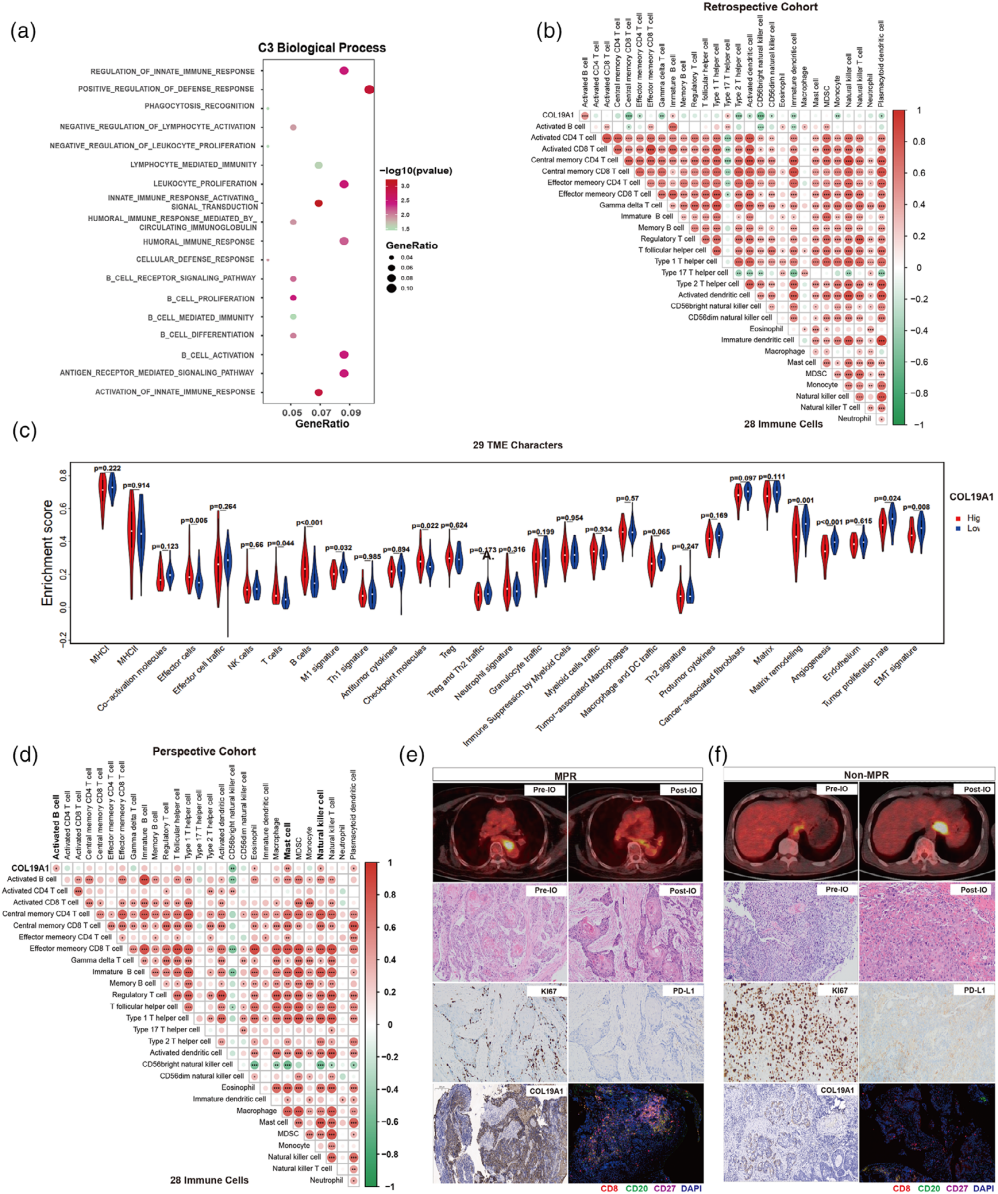
High *COL19A1* expression was associated with an inflamed immune context in esophageal squamous cell carcinoma (ESCC) patients. (a) The results of GO‐BP biological process enrichment. (b) Correlation map and heatmap analysis of the association of *COL19A1* to immune cell gene signatures of 28 immune cells (the retrospective cohort). (c) Different distribution of 29 types of immune related signatures of the C3 molecular subtype (the retrospective cohort). (d) Correlation map and heatmap analysis of the association of *COL19A1* with immune cell gene signatures of 28 immune cells (the prospective cohort). *, **, ***, and **** represent *p* < 0.05, *p* < 0.01, *p* < 0.001, and *p* < 0.0001, respectively.

The TME is associated with the efficacy of immunotherapy. In this study, patients in the retrospective cohort with the C3 subtype had distinct biological features that were related to immunotherapy responsiveness. C3‐upregulated genes (Figure 3a) were enriched for 18 immune‐related GO biological process terms, including “B cell activation,” “B cell proliferation,” “B cell receptor signaling pathway,” and “antigen receptor mediated signaling pathway” (Figure 4a). Significant differences in memory B cells (*p* < 0.0001), immature B cells (*p* < 0.05) and activated B cells (*p* < 0.05) were evident between the C3 subtype and the other subtypes (Figure S2).

Upregulated *COL19A1* gene expression was related to immune effector cells (memory B cells, immature B cells, and activated B cells) in the retrospective cohort (Figure 4b). In parallel, the infiltration level of B cells (*p* < 0.001), checkpoint molecules (*p* = 0.022), and T cells (*p* = 0.044) were greater in the *COL19A1* ^high^ group than in the *COL19A1* ^low^ group (Figure 4c). In the prospective cohort, memory B cells, immature B cells, and activated B cells were also significantly related to *COL19A1* expression (Figure 4d). We therefore evaluated the baseline expression levels of CD27 and CD20 in tumor tissues by mIF. As expected, high CD20+ and CD27+ B‐cell infiltration, as well as CD8+ T cell infiltration, were observed in the C3 subtype, with the best response to neoadjuvant immunotherapy (Figure 4e, f).

## DISCUSSION

Immunotherapy has gradually become a major focus of cancer therapy research. Recently, anti‐PD‐1/PD‐L1 antibodies have been shown to be effective treatments for recurrent and refractory ESCC patients.^15^, ^28^, ^29^ With clinical results and biomarker analysis, neoadjuvant chemoimmunotherapy has become a valid approach for ESCC patient treatment.^30^ However, tumor heterogeneity within the TME contributes to cancer progression and failure of immunotherapy.^31^, ^32^, ^33^ Thus, identification of patients who may benefit most from ICIs is of clinical significance.

Herein, we performed a systematic series of analyses to screen a panel of potential predictive biomarkers for successful treatment response. Subsequently, a “biology‐centric” method was used to precisely target candidate biomarkers. Based on transcriptome profiles from the retrospective cohort, intertumoral heterogeneity was identified by unsupervised clustering. Based on those results, 66 ESCC patients were classified into four subtypes, with immune subtype (C3) patients exhibiting better long‐term survival. Notably, subtype‐specific genes were identified as “sensitive” biomarkers, due to the immune‐activation characteristics of the C3 subtype. As a result, six genes were found to be remarkably upregulated and significantly related to clinical response. Among those, the *COL19A1* gene is rarely expressed by cancer cells but is highly expressed in amyotrophic lateral sclerosis.^34^ Li et al. reported high *COL19A1* expression in normal esophageal tissues.^35^ Further, Brodsky et al. observed that *COL19A1* ^high^ contributed to prolonged OS in gastric cancer patients.^36^ We found that high *COL19A1* expression was associated with a better prognosis for ESCC patients. Similarly, in the neoadjuvant immunotherapy cohort, we observed for the first time that *COL19A1* ^high^ was related to MPR status. As validated by IHC, *COL19A1* was predominantly expressed by ESCC malignant epithelial cells in a membrane‐anchoring manner. Further, the *COL19A1* protein has been shown to participate in the organogenesis of the esophagus as well as the maintenance of its normal structure.^37^

The TME plays a critical role in tumor development and in the response to immunotherapy of a variety of solid tumors including lung cancer,^38^ triple‐negative breast cancer, and colon cancer.^39^ Previous studies, such as that by Xu et al.^40^ implied that activated B cells are observed only in response to immunotherapy. In this study, a subset (C3) of ESCC patients in the retrospective cohort was shown to be immune‐activated and to be specifically characterized by B cell infiltration. As mentioned above, B cell accumulation was found only in patients who achieved MPR. Recent studies have found an association between enhanced B cell infiltration, apart from tertiary lymphoid structures, and an increased response to immunotherapy for various malignancies.^41^, ^42^, ^43^ In addition, this study demonstrated B cell markers to have prognostic value, of which *COL19A1*, as a B cell receptor related gene,^44^ was most related to prognosis. Our study found *COL19A1* expression to be positively related to greater immune cell infiltration (CD8+ T cells, B cells, M1 macrophages, and NK cells), which implies that *COL19A1* expression is associated with an inflamed ESCC microenvironment. An inflamed TME suggests pre‐existing immune‐specific activity and is a predictive marker of a positive response to cancer immunotherapy.^45^ Further, an inflamed microenvironment has been related to the efficacy of chemotherapy and immunotherapy.^45^, ^46^ Consequently, the predictive significance of a *COL19A1* expression pathway is reasonable to presume, in that such a pathway would reflect interaction between the immune response and malignancy. *COL19A1* may serve as a gatekeeper within the esophagus in that it putatively interacts with B cells. The mechanism of this interaction is unknown and requires further investigation.

This study has several limitations. First, participants in the prospective cohort received “real‐world” treatments that were not uniform, and therefore, our outcomes were inevitably biased. Second, this was a single‐center study that included a relatively small cohort. A larger patient population is needed to further validate *COL19A1* as a predictive biomarker for neoadjuvant chemotherapy responsiveness. Finally, the precise mechanistic basis for this study's observations has not been established and requires further intensive investigation.

The key strengths of this study are exploration of the molecular subtypes of ESCC patients, evaluation of patient subtype‐specific biological characteristics, and the construction of an immune‐related prediction model.

This study proposed, for the first time, that upregulated *COL19A1* is a biomarker that can predict the response to neoadjuvant immunotherapy by ESCC patients. We also found *COL19A1* expression to be associated with an inflamed ESCC microenvironment. Altogether, these findings provide valuable insight into the predictive value of *COL19A1* as a means to precisely screen and predict disease outcomes in ESCC patients who may benefit from neoadjuvant immunotherapy.

## AUTHOR CONTRIBUTIONS

Concept and design: JM T; resources: G‐B Q; supervision, JM T and G‐B Q; writing‐original draft and data curation: J‐H L; investigation: J‐H L and J‐Z L; project administration: J‐Z L; validation: Q‐H Q; methodology and formal analysis: C‐B Z and WW L; data curation: C‐B Z, W‐W L, Q‐L, ZH‐H; software and visualization: XR X; final approval of manuscript: all authors; accountable for all aspects of the work: all authors.

## CONFLICT OF INTEREST STATEMENT

Weiwei Li, Changbin Zhu, Qian Li, and Zhan Huang were employed by Amoy Diagnostics Co., Ltd. None of the other authors have any conflicts of interest to declare.

## Supporting information


**APPENDIX S1.** Supplementary Materials.

**FIGURE S1.** Estimation of ESCC clusters based on transcriptome profile. (A) Molecular functional portrait (potential target genes, signaling pathways, and cellular processes related to each of 29 TME gene expression signatures created by Bagaev et al.) of C3 subtype. (B‐G), Comparison of clinical stage, N stage, metastatic sites, distance of metastases, vascular invasion, and neuro‐invasion distributions among different molecular subtypes. TME: tumor microenvironment. Reference: Bagaev A, Kotlov N, Nomie K, Svekolkin V, Gafurov A, Isaeva O, et al. Conserved pan‐cancer microenvironment subtypes predict response to immunotherapy. Cancer Cell. 2021;39 (6):845–865 e847. 10.1016/j.ccell.2021.04.014.

**FIGURE S2.** The distribution of B cells and mast cells in non‐MPR and MPR patients of the C3 subtype. Boxplots indicating the fraction of 28 types of immune‐related cells among the four molecular subtypes from the retrospective cohort. MPR: major pathological remissions.

## Data Availability

The datasets used and analyzed during the current study are available from the corresponding author upon reasonable request.
