# Supplementary material for: COL19A1 is a predictive biomarker for the responsiveness of esophageal squamous cell carcinoma patients to immune checkpoint therapy
Source: Thorac Cancer. 2023 Apr 2;14(14):1294–305. doi: 10.1111/1759-7714.14873 (PMC10175035; doi:10.1111/1759-7714.14873)
Supplement: Supplementary file 1 — APPENDIX S1. Supplementary Materials. [file TCA-14-1294-s003.docx]

**Appendix S1**

**Supplementary Information**

1. **Supplementary Materials**

Immunohistochemistry (IHC) was conducted using primary antibodies reactive with; Ki-67 (dilution: 1: 200, DAKO, M7240), PD-L1 (dilution: 1: 2000, DAKO 22C3, M3653), and COL19A1 (dilution: 1: 200, Sigma-Aldrich, HPA042422). Multiplex Immunofluorescence (mIF) were performed with primary antibodies reactive with CD8 (dilution 1:100), CD20 (dilution 1:100), and CD27 (dilution: 1:100).

AmoyDx® FFPE RNA Extraction Kits, for extraction of total RNA, were obtained from Amoy Diagnostics Co., Ltd. (AmoyDx), Xiamen, China (Cat. # 8.02.24101X036G). Quantus RNA HS Assay Kit for the quantification of RNA concentration was purchased from Promega Biotech Co., Ltd., Beijing, China (Cat. # E2670/E3310). NEBNext® Ultra™ II Directional RNA Library Prep Kits for Illumina® were from New England Biolabs, Inc. (NEB, Cat. #E7760L). DNA/RNA HS Kits for evaluation of fragment length was from Agilent Technologies Co., Ltd. [Beijing, China (Cat. # 5067-1504/5067-1511)]. Opal Polaris 5-Color Automation IHC Detection Kits were from Akoya Biosciences (Menlo Park, CA, USA).

1. **Supplementary Figures**

**Figure S1**. Estimation of ESCC clusters based on transcriptome profile.

(A) Molecular functional portrait (potential target genes, signaling pathways, and cellular processes related to each of 29 TME gene expression signatures created by Bagaev *et al*.) of C3 subtype. (B-G), Comparison of clinical stage, N stage, metastatic sites, distance of metastases, vascular invasion, and neuro-invasion distributions among different molecular subtypes. TME: tumor microenvironment. Reference: Bagaev A, Kotlov N, Nomie K, Svekolkin V, Gafurov A, Isaeva O, et al. Conserved pan-cancer microenvironment subtypes predict response to immunotherapy. Cancer Cell. 2021;39(6):845-865 e847. <https://doi:10.1016/j.ccell.2021.04.014>.


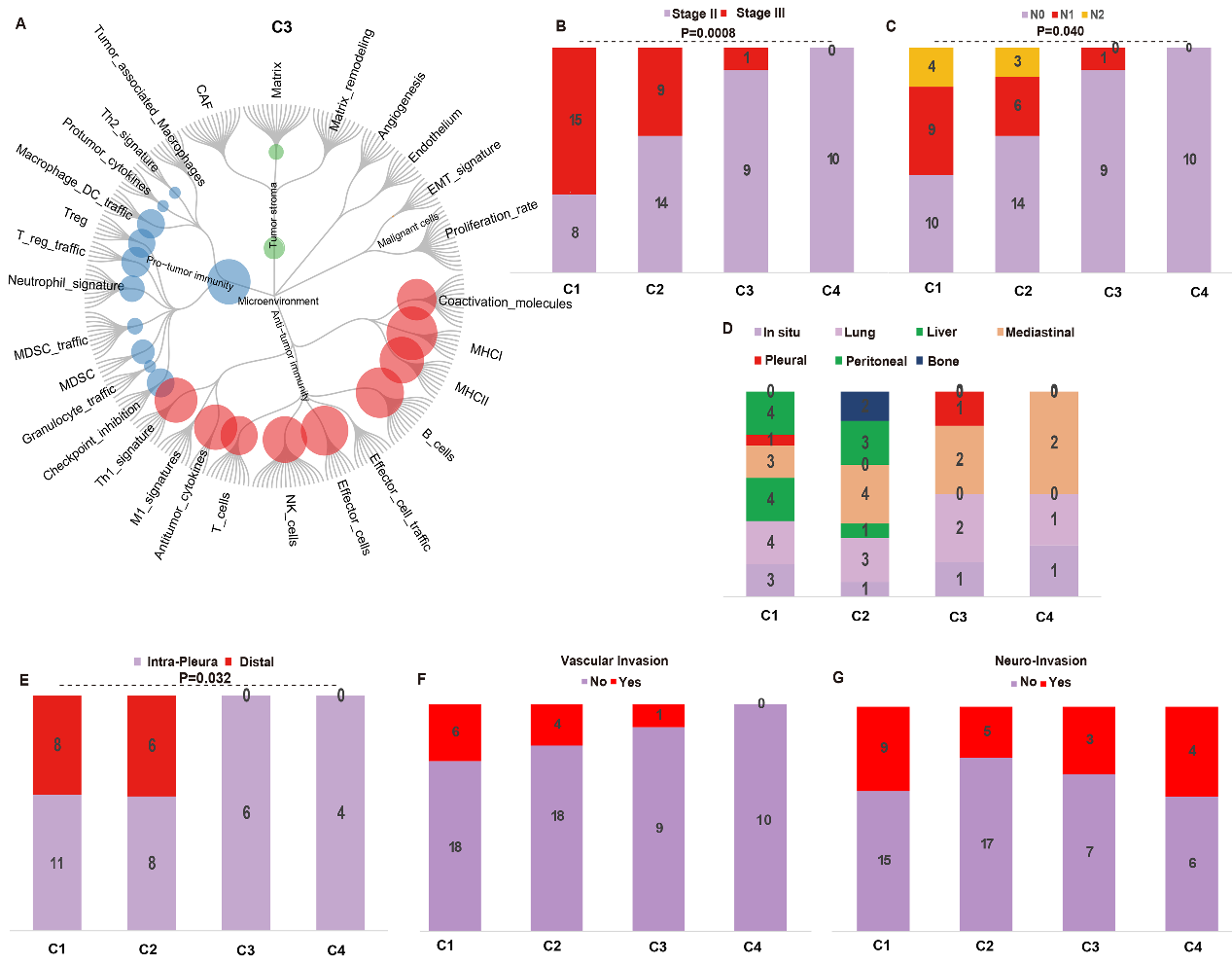


**Figure S2.** The distribution of B cells and mast cells in non-MPR and MPR patients of the C3 subtype. Boxplots indicating the fraction of 28 types of immune-related cells among the four molecular subtypes from the retrospective cohort. MPR: major pathological remissions.

**
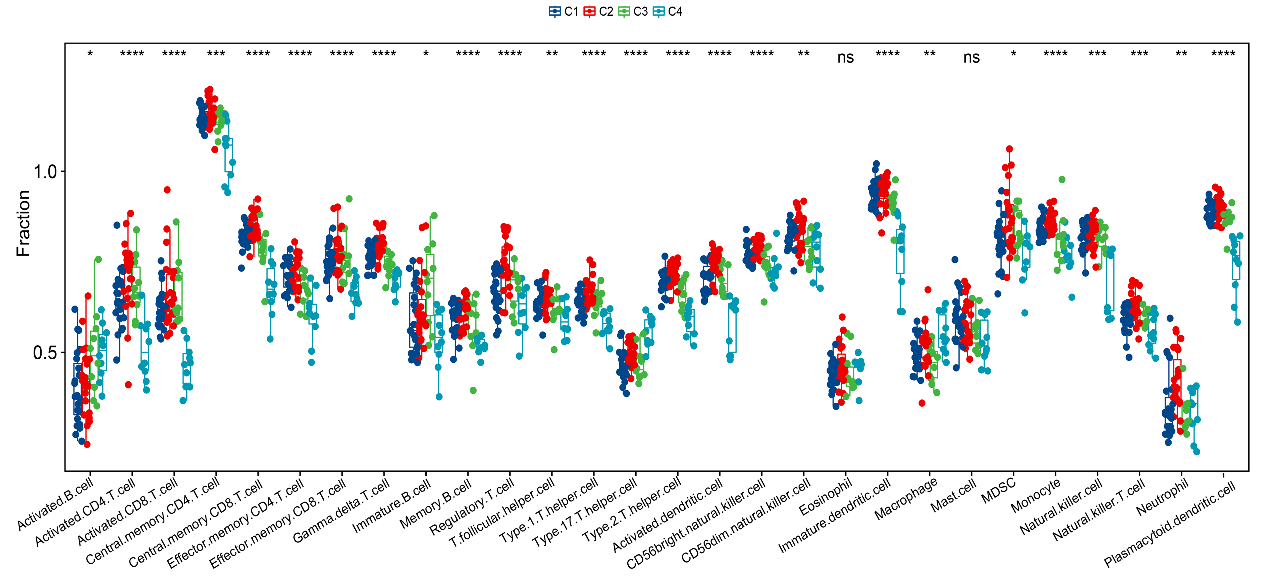
**
