# Supplementary figures and images for: COL19A1 is a predictive biomarker for the responsiveness of esophageal squamous cell carcinoma patients to immune checkpoint therapy
Source: Thorac Cancer. 2023 Apr 2;14(14):1294–305. doi: 10.1111/1759-7714.14873 (PMC10175035; doi:10.1111/1759-7714.14873)

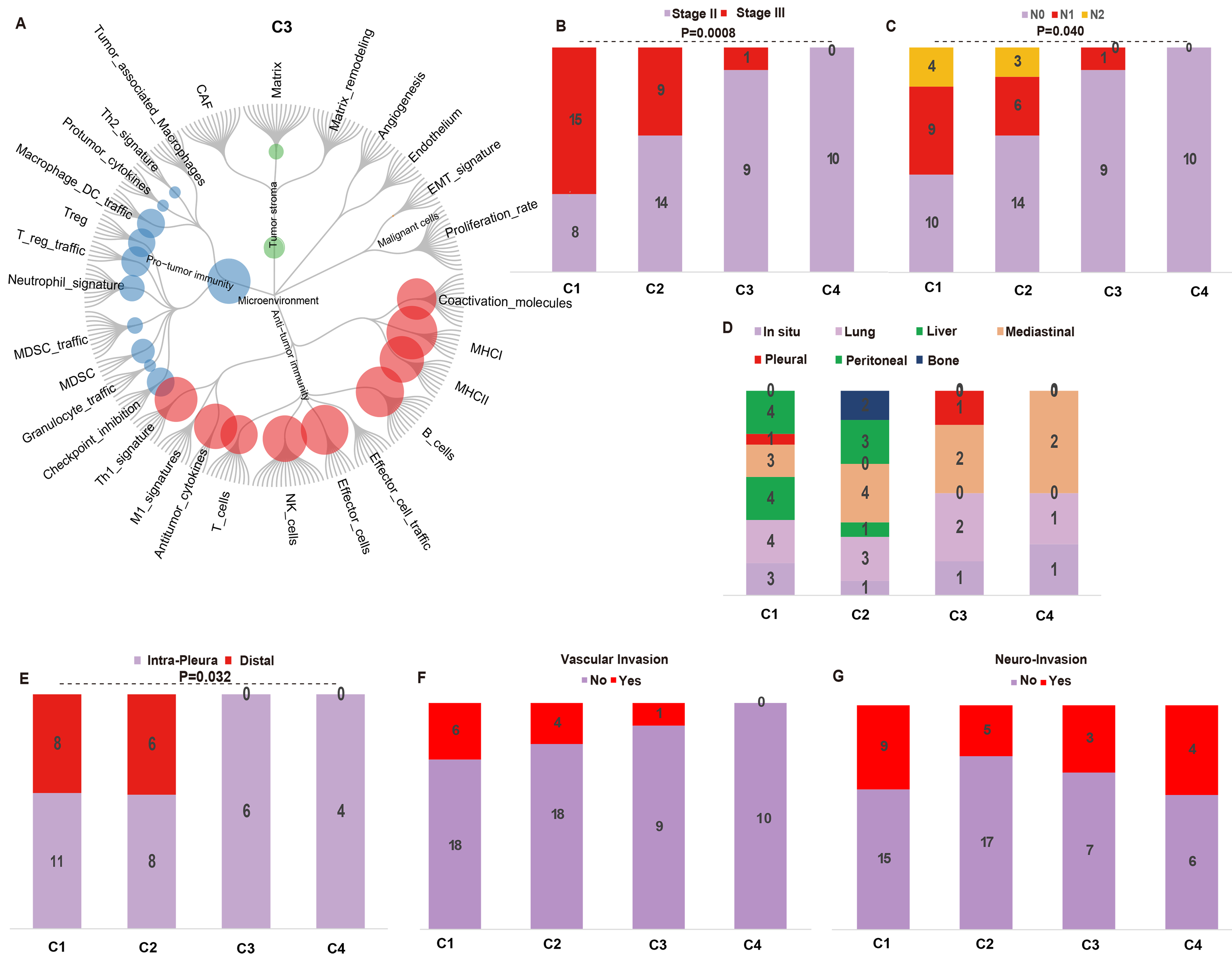

Supplement: Supplementary file 2 — FIGURE S1. Estimation of ESCC clusters based on transcriptome profile. (A) Molecular functional portrait (potential target genes, signaling pathways, and cellular processes related to each of 29 TME gene expression signatures created by Bagaev et al.) of C3 subtype. (B‐G), Comparison of clinical stage, N stage, metastatic sites, distance of metastases, vascular invasion, and neuro‐invasion distributions among different molecular subtypes. TME: tumor microenvironment. Reference: Bagaev A, Kotlov N, Nomie K, Svekolkin V, Gafurov A, Isaeva O, et al. Conserved pan‐cancer microenvironment subtypes predict response to immunotherapy. Cancer Cell. 2021;39 (6):845–865 e847. 10.1016/j.ccell.2021.04.014. [file TCA-14-1294-s002.tif]

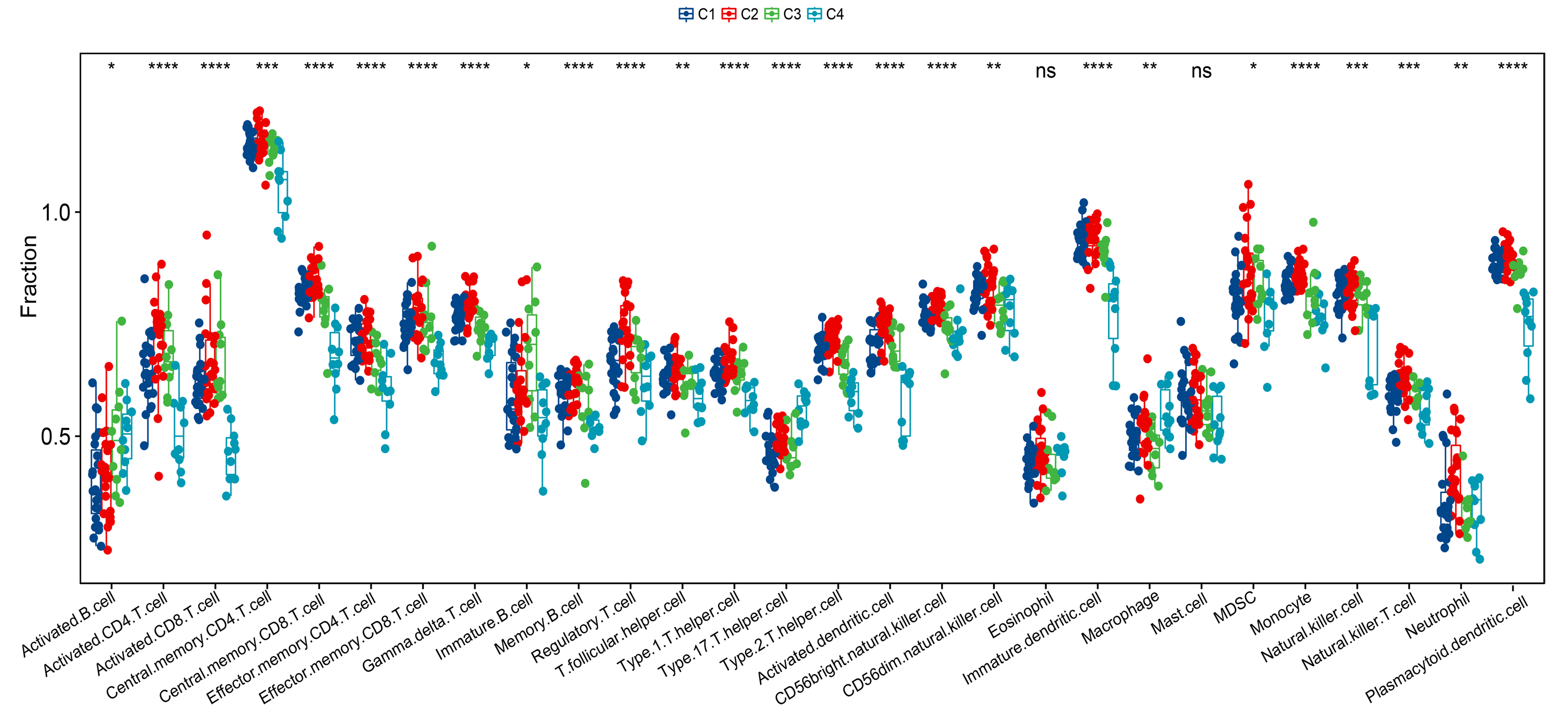

Supplement: Supplementary file 3 — FIGURE S2. The distribution of B cells and mast cells in non‐MPR and MPR patients of the C3 subtype. Boxplots indicating the fraction of 28 types of immune‐related cells among the four molecular subtypes from the retrospective cohort. MPR: major pathological remissions. [file TCA-14-1294-s001.tif]
